# Leveraging relatedness‐based measures in people with language disorders: A scoping review

**DOI:** 10.1111/jnp.12405

**Published:** 2024-12-16

**Authors:** Logan A. Gaudet, Lena Rybka, Emmanuel Mandonnet, Emmanuelle Volle, Marion Barberis, Roel Jonkers, Adrià Rofes

**Affiliations:** ^1^ Center for Language and Cognition Groningen (CLCG) University of Groningen Groningen The Netherlands; ^2^ Research School of Behavioural and Cognitive Neurosciences (BCN) University of Groningen Groningen The Netherlands; ^3^ FrontLab at Institut du Cerveau (ICM) Sorbonne Université Paris France; ^4^ Charité‐Universitätsmedizin Berlin Klinik für Neurochirurgie Berlin Germany

**Keywords:** relatedness‐based measures, scoping review, semantic networks, semantic system, verbal fluency

## Abstract

Understanding lexico‐semantic processing is crucial for dissecting the complexities of language and its disorders. Relatedness‐based measures, or those which investigate the degree of relatedness in meaning between either task items or items produced by participants, offer the opportunity to harness novel computational and analytical techniques from cognitive network science. Recognizing the need to deepen our understanding of lexico‐semantic deficits through diverse experimental and analytical approaches, this review explores the use of such measures in research into language disorders. A comprehensive search of four electronic databases covering publications from the last 11 years (October 2013–September 2024) identified 38 original experimental studies employing relatedness‐based measures in populations with language disorders or other neurological conditions. Articles were examined for the types of tasks used, populations studied, item selection methods and analytical approaches. The predominant use of category fluency tasks emerged across studies, with a notable absence of relatedness judgement tasks or comparable paradigms. Commonly discussed populations included individuals with post‐stroke aphasia, mild cognitive impairment and schizophrenia. Analytical methods varied significantly, ranging from more traditional approaches of clustering and switching to more sophisticated computational techniques. Despite the evident utility of category fluency tasks in research and clinical settings, the review underscores a critical need to diversify experimental paradigms and probe lexico‐semantic processing in a more multifaceted manner. A broadened approach in future language disorder research should incorporate innovative analytical techniques, investigations of neural correlates and a wider array of tasks employing relatedness‐based measures already present in healthy populations.

## INTRODUCTION

Relatedness‐based measures allow for an investigation of the association strength between distinct items in the semantic system, either using participant behaviour to estimate the relatedness in meaning between task items (e.g., given the words ‘cat’ and ‘dog’, participants are asked the degree to which the two relate to one another, using a scale), or by investigating the relatedness in meaning between items produced by participants (e.g., a participant produces ‘cat’ and ‘dog’ in a fluency task, and the degree of semantic relatedness between the two is considered as part of an experimental measure). The primary motivation behind this review is to understand which such measures have been employed in studies of individuals with language disorders, to whom and how they have been used, and what the general findings of such studies have been. This work is relevant because, to the best of our knowledge, the number of experimental paradigms that have used these measures in people with language disorders is much smaller than those employed in healthy people. Relatedness‐based measures enable the use of computational methods from cognitive network science and may offer novel insight into mild language impairments as well as the underlying nature of lexico‐semantic organization (Kumar, 2021; Reilly et al., 2023; Whitworth et al., 2014). Hence, this review aims to elucidate a gap in the literature and to offer ways forward for research.

The semantic system is a core component of cognition and linguistic processing, as information is retrieved from this system in response to auditory/written words, ideas or concepts (Binder et al., 2009; Jones et al., 2015; Whitworth et al., 2014). The semantic system is central to language processing (Ellis & Young, 1996; Patterson & Shewell, 1987; Whitworth et al., 2014), and fundamental to our understanding of the neuroarchitecture that supports linguistic processing (Binder & Desai, 2011; Chang et al., 2015; Patterson et al., 2007). Indeed, semantic information is necessary for both the production and comprehension of language (Whitworth et al., 2014). It is of particular clinical relevance to understand and preserve the semantic system, as damage may significantly reduce linguistic abilities and quality of life (Dvorak et al., 2021).

Deficits to the semantic system are observed in people with a number of neurological conditions, including stroke, neurodegeneration, autoimmune neurological disease and brain infection, among others (Gorno‐Tempini et al., 2011; Hodges et al., 2000; Lambon Ralph et al., 2010; Reilly et al., 2011; Rofes et al., 2021; Rogers & Friedman, 2008; Rook et al., 2023; Wilson et al., 2017). Semantic deficits are also reported among individuals who have undergone awake brain surgery for the excision of primary brain tumours in the left hemisphere (Campanella et al., 2009; Rofes et al., 2018; Satoer et al., 2018). However, the semantic system cannot be observed directly. As a multifaceted and complex system, its properties and organizational structure can only be deduced through carefully designed experimental paradigms, with different tasks tapping into different aspects of lexical semantics (Kumar, 2021). As a result, a multitude of paradigms have been documented in the literature, each purporting to reveal varying aspects of the semantic system. We will first discuss some of the more common experimental paradigms (e.g., priming, picture‐word interference and non‐verbal association tasks) before characterizing those tasks that lend themselves to relatedness‐based measures (e.g., relatedness judgement tasks, category verbal fluency with semantic similarity ratings).

### Experimental paradigms

Firstly, investigations of priming (also called ‘semantic priming’), in which the processing of a word is facilitated by a preceding related word (e.g., the ‘prime’ word *yellow* might facilitate the processing of the ‘target’ word *banana*), are perhaps the most well established in assessing lexico‐semantic processing (McNamara, 2005; Neely, 1991). This is not without good reason: priming tasks have a relatively straightforward experimental design, are backed by decades of ongoing research (e.g., Hutchison, 2003; Hutchison et al., 2013; Meyer & Schvaneveldt, 1971), and can unveil crucial insights about the structure of the semantic system (for overviews see Kumar, 2021; McNamara, 2005). This type of study has provided support for several models of semantics, perhaps chief among them being spreading activation models (e.g., Collins & Loftus, 1975). Support has also been proposed for distributed, feature‐based, and associative network models, with elements of multiple models often being integrated for a more comprehensive understanding of the semantic system (e.g., Hutchison, 2003; McRae, 2004; Plaut & Booth, 2000). Advocating for one perspective or another is outside of the scope of this article. However, in general terms, these models agree on the concept of an interconnected semantic system and the role of activation in lexico‐semantic processing, and disagree on the mechanisms underlying these processes and the structure of semantic representations (Kumar, 2021; Reilly et al., 2023). In other words, while these models collectively acknowledge an intricate interconnection of word meanings, akin to a vast network, they differ in their views on how this network operates and how our brains organize these connections.

At the clinical level, priming studies have been used to characterize lexico‐semantic deficits in different neurological populations: while individuals with post‐stroke aphasia and impaired lexical comprehension have shown evidence of significant priming effects (Blumstein et al., 1982), the opposite has been found for individuals with post‐stroke aphasia and impaired lexical production (Del Toro, 2000). However, more recent research has conversely suggested that priming is normal and unimpaired in most people with post‐stroke aphasia, with authors calling into question the utility of such tasks in assessing lexico‐semantic impairment (Dyson et al., 2020). Additionally, drawing conclusions about the degree of relatedness between primes and targets can be unreliable at the item level (Heyman et al., 2018), which may impede the ability of priming studies to elucidate more fine‐grained details of semantic structure. For example, while a priming study may indicate that the words ‘yellow’ and ‘banana’ are related, it is difficult to objectively gauge whether this relation is greater than that between ‘sweet’ and ‘banana’.

A second category of experimental paradigm is picture–word interference experiments (also known as ‘semantic interference’ paradigms). In these tasks, participants are asked to name pictures while ignoring potential distractor words (e.g., Roelofs & Piai, 2017). Such studies can shed light on how lexico‐semantic relationships impact both linguistic and attentional processes (e.g., Scaltritti et al., 2015), and when coupled with magnetoencephalography (MEG), have provided evidence for the neuroplasticity of language in brain tumour patients (Piai et al., 2020). Object and action naming tasks, in which participants produce nouns or verbs represented by the presented stimuli, are an additional common paradigm (e.g., Deloche & Hannequin, 1997). Naming tasks rely on multiple aspects of language, including not only lexico‐semantic access and retrieval but also object recognition and articulation (Rofes & Mahon, 2021). Such naming paradigms have contributed considerably to neurolinguistic research and to perspectives on lexico‐semantic processing (Bozeat et al., 2000; Duong et al., 2006; Piai & Eikelboom, 2023). They have also contributed to diagnostic and intraoperative procedures (Bozeat et al., 2000; Ntemou et al., 2023; Rofes & Miceli, 2014).

A third category of experimental paradigm tapping into semantic cognition is non‐verbal association tasks, used frequently as clinical measures of semantic decline (Klein & Buchanan, 2009). In the Pyramids and Palm Trees Test (PPTT), participants are presented with triads of object images, and are asked to select which of the two lower objects is most closely associated with the test item above (Howard & Patterson, 1992). Performance on the PPTT relies on visual object recognition, semantic retrieval processes and making associations between object representations (Callahan et al., 2010; Klein & Buchanan, 2009). The Kissing and Dancing Test (KDT) is analogous to the PPTT but differs in that the images represent actions, rather than objects (Bak & Hodges, 2003). Triadic comparison tasks, which involve selecting the least similar item from a set of three, are also used to assess changes in semantic memory and are included in some well‐established cognitive batteries for Alzheimer's disease (AD, e.g., Shankle et al., 2009). Such tasks have contributed to our understanding of the neural substrates of lexico‐semantic processing: for example, pairing direct electrical stimulation with the PPTT has suggested that impairments in the dorsolateral prefrontal cortex and the right inferior fronto‐occipital fasciculus could lead to deficits in non‐verbal semantic cognition (Herbet et al., 2017, 2018). Comparable action‐based association tasks have demonstrated that lesions in left‐hemisphere regions, including the inferior frontal gyrus, ventral precentral gyrus, anterior insula, ventral postcentral gyrus, supramarginal gyrus and posterior middle temporal gyrus, are associated with action‐related semantic deficits (Kemmerer et al., 2012).

However, tasks like the PPTT may not be sensitive to mild semantic deficits (Adlam et al., 2010). Behavioural measures in these tasks are typically limited to accuracy and response times (Fergadiotis et al., 2010), with responses being inherently binary as predetermined by task design. These design features may restrict the capacity of these tasks to unveil subtle variations in item‐level associations or more complex aspects of lexico‐semantic organization (Klein & Buchanan, 2009; Ohman et al., 2022).

### Relatedness‐based measures

As alluded to in the opening paragraph, some authors have recently stressed that new methods are necessary to disentangle competing descriptions of the semantic system and input/output lexica, and to continue describing lexico‐semantic deficits in clinical populations. The advent of computational techniques from fields such as cognitive network science has given rise to researchers using word networks (semantic, phonological, or both in the case of multiplex networks) and visualizations of semantic space (e.g., Bose et al., 2017; Castro & Stella, 2019; Kenett et al., 2016; Nevado et al., 2021; Nour et al., 2023; Vitevitch, 2022; Zemla & Austerweil, 2018). Within these types of studies, crucial elements of lexico‐semantic processing can be investigated by either implicitly or explicitly asking participants to make associations between distinct concepts or words (e.g., how often is ‘yellow’ mentioned when people are given the word ‘banana’ or how similar are ‘yellow’ and ‘banana’ given a specific scale). This is done by administering tasks that allow researchers to characterize or quantify those associations, for example, verbal fluency tasks (e.g., Nour et al., 2023; Zemla & Austerweil, 2019) and relatedness judgement tasks (e.g., Cosgrove et al., 2023; Ovando‐Tellez, Kenett, et al., 2022).

In the present review, we will refer to the measures in such tasks as relatedness‐based measures, operationalized as those which either use participant behaviour to estimate the relatedness in meaning between task items (e.g., relatedness judgement task), or investigate the relatedness in meaning between items produced by participants (e.g., verbal fluency). In other words, participant behaviour is an essential component in determining either the degree of subjective relatedness between items, or the items between which relatedness is to be investigated. These measures thus offer the opportunity to investigate more directly the association strength that exists between distinct items within the semantic system and/or the lexica of the participant, compared to the experimental paradigms we discussed above, in which predetermined association measures reflect the semantic relatedness between stimuli independent of the participant. While they are not necessarily superior, tasks with relatedness‐based measures are in this way different from the aforementioned paradigms of priming, picture–word interference, naming and the PPTT which typically investigate effects that are secondary to association strength or relatedness (e.g., reaction time, accuracy; Bürki & Madec, 2022; Higby et al., 2019; Klein & Buchanan, 2009; McNamara, 2005; Moritz‐Gasser et al., 2012).

Firstly, one category of tasks with relatedness‐based measures are relatedness judgement tasks (RJT). In a task such as the RJT of Bernard et al. (2019), participants are presented with pairs of words and are asked to indicate a subjective association rating on a scale from 0 to 100, indicating the degree to which they perceive the two words to be related in meaning. For example, the thematic association between *nose* and *flower* may elicit a higher rating than that which would be elicited by *nose* and *existence*, where a clear association may not be present. From these tasks, semantic networks can be generated with the aim of reflecting the semantic system's underlying structure, allowing for both the detailed modelling of individual networks as well as comparisons between them (Castro & Siew, 2020; De Deyne et al., 2017; He et al., 2021; Siew et al., 2019). Such networks consist of nodes representing concepts or words in the semantic system, and edges representing the semantic similarity between nodes (Collins & Loftus, 1975; Siew et al., 2019). Graph theory analyses of networks derived from a variety of experiment paradigms have been used to show structural differences in the lexico‐semantic organization of several neurological populations, including people with AD, autism‐spectrum disorder and schizophrenia, as well as in late talker children and healthy ageing (Beckage et al., 2011; Cosgrove et al., 2023; Kenett et al., 2016; Paulsen et al., 1996; Stam et al., 2007). The potential to generate semantic networks from tasks with relatedness‐based measures is one of the primary motivations for this review.

A second category of relatedness‐based measures are those which can be extracted from verbal fluency tasks. These measures are in a sense less explicit than those that can be derived from relatedness judgement tasks. Category fluency tasks demand the production of as many words from a particular semantic category as possible (e.g., animals) within a predefined time limit (e.g., Lezak et al., 2012; Rofes et al., 2023; Stolwyk et al., 2015). Aside from asking that the words fall into the animal category, participants are not explicitly told to produce words with any further level of association with one another. However, highly related words are typically produced in a sequential manner, grouped by association strength and shared semantic properties (i.e., ‘clusters’, e.g., farm animals, insects, aquatic animals, etc.) (Sung et al., 2012; Thiele et al., 2016; Troyer et al., 1997). Search models such as the *optimal foraging theory* describe how these clusters and other related sequence‐level aspects of fluency task responses can reflect exploration within the lexico‐semantic space (Hills et al., 2015), and the implicit associations revealed by differing fluency task responses can thus reflect differences in lexico‐semantic access or organization (Hills et al., 2012; Ovando‐Tellez, Benedek, et al., 2022). Category fluency tasks are also suitable for the generation of semantic networks (for an overview of prominent methods, see Zemla, 2022). In contrast to relatedness judgement tasks, where nodes in the network are predetermined by task design and edges are derived from participant behaviour (e.g., Benedek et al., 2017), nodes and the associations between them can instead be determined based on the order and co‐occurrence of items in fluency responses (e.g., Cosgrove et al., 2021; Goñi et al., 2011; Kenett et al., 2013; Zemla & Austerweil, 2018). However, while relatedness‐based measures may be an invaluable tool in informing our knowledge about the lexico‐semantic system, making use of novel computational techniques, and making clinical assessments in cases of impairment, we are unaware of any reviews to date which have attempted to provide an overview of those tasks which employ them.

To the best of our knowledge, there has not yet been an effort to compile a unified knowledge base or set of terminology surrounding tasks with relatedness‐based measures, their respective contributions to our understanding of the semantic system, or their applicability in different neurological populations. It is the aim of the present review to address this gap for these types of tasks/measures, particularly for work published over the last 11 years. It is hoped that the conclusions drawn from this review will aid in informing future research, provide a concise overview to researchers and clinicians, and highlight critical gaps that are worth exploring in future research.

### The present study

We aim to provide a comprehensive overview of tasks employing relatedness‐based measures as they are used in populations with language disorders, and will seek to describe the experimental paradigms, test items and item‐selection methods used in each. Due to the heterogeneity of terms referring to relatedness‐based measures in contemporary literature, the present review, as stated previously, will operationalize these measures as those which investigate the degree of relatedness in meaning between either test items or items produced by participants.

Specifically, we will address the following research questions:

1. What tasks employing relatedness‐based measures have been reported in adults with language disorders?
2. What types of language disorders and neurological pathologies are discussed?
3. What types of items are used in these tasks, and how are those items selected?
4. What analysis methods are used in these tasks?

We expect that studies employing relatedness measures between items produced by participants will largely comprise category fluency tasks and comparable verbal generation paradigms, while studies investigating relatedness in meaning between task items will be similar in design to the aforementioned relatedness judgement tasks. We additionally expect that studies will be found whose relatedness‐based paradigms may not yet be familiar to the authors. We anticipate category fluency studies with relatedness measures to be common among those populations who frequently perform such tasks clinically (e.g., post‐stroke aphasia, neurodegeneration). In the case of tasks where relatedness in meaning between task items is assessed, we expect to find information about how these items were selected, in particular the linguistic variables for which task conditions and stimulus lists may have been balanced (e.g., frequency, age of acquisition, concreteness, relatedness). We anticipate analytical techniques comparable to those that have been applied in healthy populations (e.g., Benedek et al., 2017; Ovando‐Tellez, Kenett, et al., 2022) to also be present in clinical linguistic research, and that novel computational measures for verbal fluency will be revealed.

## METHODS

A scoping review of the literature was conducted using the following databases: PubMed, Embase, PsycInfo and Web of Science (Core collection). Searches were conducted in October of 2023 and October of 2024, with queries and search strategies formulated for each database. The search strategy as applied in PubMed is available in Table 1, while comparable search strings for each of the databases can be found in Table S1. A protocol for the scoping review following the PRISMA‐P guidelines (Moher et al., 2015) was published and pre‐registered on PROSPERO (https://www.crd.york.ac.uk/prospero/display_record.php?ID=CRD42024508094). A scoping review was chosen due to the anticipated heterogeneity of study populations and analytical methods, and because the primary purpose of this review is to examine the state of research in this domain and identify gaps in the research knowledge base (Colquhoun et al., 2014; Munn et al., 2018; Peters et al., 2015).

**TABLE 1:** Search string used for article retrieval in PubMed.

Database	Search string	Limit
PubMed (https://pubmed.ncbi.nih.gov/)	(‘Language Disorders’[Mesh] OR ‘Aphasia’[Mesh] OR ‘Brain Neoplasms’[Mesh] OR ‘Communication Disorders’[Mesh]) AND (‘Word Association Tests’[Mesh] OR ‘verbal fluency’[tiab] OR ‘semantic fluency’[tiab] OR ‘category fluency’[tiab] OR (‘cluster*’[tiab] AND ‘switch*’[tiab]) OR ‘semantic network*’[tiab] OR ((‘related*’[tiab] OR ‘association’[tiab] OR ‘associative’[tiab] OR ‘semantic’[tiab]) AND (‘task’[tiab] OR ‘test’[tiab] OR ‘judgment’[tiab] OR ‘judgement’[tiab] OR ‘evaluation’[tiab])))	October 2013 to September 2024

**FIGURE 1:**
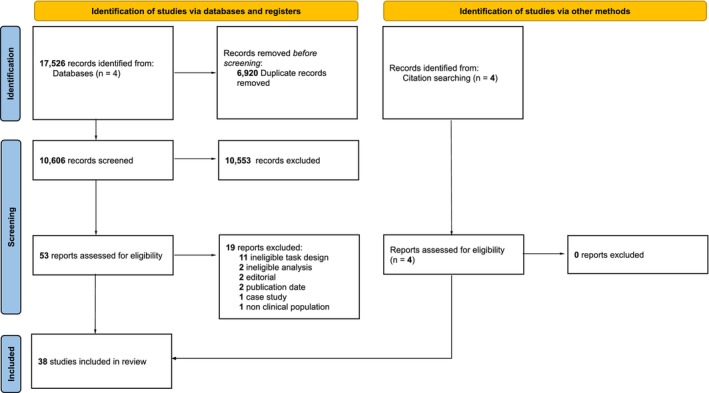
PRISMA‐P flow diagram of the literature search and screening process.

The selection process for article inclusion is illustrated in Figure 1. The criteria for inclusion in the present review was as follows: (1) original experimental studies published within the 11 years prior to the review (October 2013 to September 2024); (2) studies for which the participants were adults with linguistic deficits and/or neurological disorders (e.g., stroke, brain tumours, brain infections, neurodegeneration, autoimmune neurological diseases); (3) studies which investigate the degree of relatedness in meaning between either task items or items produced by the participants (e.g., category verbal fluency, semantic association tasks, and other tasks with relatedness‐based measures) and (4) studies published in English, French, German and any other language for which a proficient member of the research team could be found (e.g., Catalan, Dutch, Italian, Spanish, Portuguese). Reviews, meta‐analyses, case studies and non‐experimental studies were excluded. Studies were also excluded if they included tasks in which participants produced related items, but the relationships between these items were not considered in the analysis (e.g., where category fluency tasks were used as part of a larger cognitive battery and only the total score was considered). Similarly, studies were excluded if they included tasks in which participants made relatedness judgements, but either these judgements were not considered in the analysis, or performance on this task was not the focus of any part of the article. Studies where inter‐item relatedness was only determined prior to performance on the task (i.e., the dependent variables of interest were measures secondary to relatedness) were also excluded. This included synonym judgement tasks (e.g., Dubé et al., 2014), priming tasks (e.g., Howells & Cardell, 2015) and tasks that used predetermined multiplex network properties to predict performance on other tasks (e.g., Castro & Stella, 2019). Such tasks would be included if they were analysed in such a way that inter‐item relatedness could be investigated based on participant behaviour.

It should be noted that the search strategy was not specifically designed to capture relevant studies in psychiatric populations. However, due to an apparent tendency for novel computational techniques to be used in this field (e.g., Nour et al., 2023), and ongoing debate regarding the distinction between psychiatric and neurological disorders (Crossley et al., 2015; David & Nicholson, 2015), it was decided that relevant papers featuring psychiatric populations and captured by the present search strategy would be included. Likewise, studies in other populations characterized by linguistic differences, and not necessarily deficits, would be included so long as they appeared among the search results (e.g., autism).

In addition to electronic database searches, the reference lists of those articles whose full texts were selected for inclusion were screened for further relevant studies. Study authors were to be contacted by email in the case that otherwise inaccessible papers passed the title and abstract screening phase, with data extraction proceeding without these papers only in the case that no response was received after 2 weeks. This was not the case for any of the identified studies.

Titles and abstracts retrieved from database searches were exported into the reference manager Endnote. Following deduplication, titles and abstracts were uploaded to the collaborative systematic review platform Rayyan (Ouzzani et al., 2016; available at https://www.rayyan.ai/). Two independent reviewers assessed the titles and abstracts for eligibility. The independent reviewers were blinded to one another's decisions, with disagreements regarding eligibility being resolved through further discussion and examination relative to the pre‐established inclusion and exclusion criteria. Any persisting disagreements were resolved by the decision of a third reviewer (i.e., the senior author), and the full texts of all remaining articles following this screening phase were reviewed by the first author.

The following information was extracted from all full‐text articles selected for inclusion: general publication information (e.g., journal, publication date, authors), participant characteristics (e.g., neurological population, sample size, age, education in years), experimental groups (e.g., mild vs. severe aphasia, patient vs. control), type of task used (e.g., category fluency, relatedness judgement task), task paradigm details (e.g., method of presentation, data recorded, inter‐stimulus intervals, task length, the presence of filler stimuli), analytical methods applied, type and number of items used in the task (e.g., images, text, auditory stimuli), method of test item pre‐selection (if applicable) and type of neuroimaging information either concurrent with or analysed in relation to the task (if applicable).

## RESULTS

A total of 38 relevant studies were identified in the present review (see Figure 1). A summary of the identified papers, along with their populations, relatedness‐based tasks, analytical measures and theoretical contributions are presented in Table 2.

**TABLE 2:** Summary of identified articles and relatedness‐based measures.

Study	Clinical population	Experimental groups	Task(s) with relatedness‐based measures	Semantic clustering method	Inter‐item relatedness measures	Other fluency measures	Theoretical contribution
Altun et al., 2024	Relapsing–remitting multiple sclerosis (RRMS)	RRMS, control	Category fluency (animals), 60s	Troyer et al., 1997	cluster size *switches* ^a^	*total score* ^a^ Fluency Difference Score (FDS)	Similar cluster size reflects preserved verbal memory and word recall in RRMS, while increased switches reflect impaired cognitive flexibility. Relatedness measures in category fluency can discern verbal memory/word recall vs. cognitive flexibility in people with RRMS
Bose et al., 2017	Post‐stroke aphasia (left hemisphere)	Aphasia, control	Category fluency (animals), 60s	Troyer et al., 1997	*Cluster size* ^a^ *switches* ^a^ change in cluster size over 15 s intervals *change in switches over 15 s intervals* ^a^	*Total score* ^a^ *within cluster pause duration* ^a^ *switch duration* ^a^ *score over 15 s intervals* ^a^	Impaired performance in aphasia is mainly due to lexical retrieval processes (smaller clusters and slower retrieval) but also partly due to executive components of the task (more switches and longer switch duration). Relatedness measures in category fluency allow the study of lexical retrieval and executive functions in people with post‐stroke aphasia
Bose et al., 2022	Post‐stroke aphasia (left hemisphere)	Aphasia, control	Category fluency (animals), 60s	Troyer et al., 1997	Cluster size *switches* ^a^	*Total score* ^a^ *within cluster pause duration* ^a^ *switch duration* ^a^ *1st‐RT* ^a^ Sub‐RT *FDS* ^a^	Impaired performance in aphasia is a result of difficulties with both lexical (total score, 1st‐RT, within cluster pause duration) and executive (switches, switch duration, FDS) components of the task. Relatedness measures in category fluency allow the study of lexical retrieval and executive functions in people with post‐stroke aphasia
Carpenter et al., 2021	Bilingual aphasia (either hemisphere; post‐stroke, TBI, or tumour)	Bilingual aphasia, bilingual control	Category fluency (animals, clothing, food, transportation), 60s, in 4 language conditions: L1, L2, forced switch L1/L2, self‐switch L1/L2	Troyer et al., 1997	*Cluster size* ^a^ *switches* ^a^	*Total score* ^a^ *FDS* ^a^	Switches are more sensitive to increased control demands than clusters. Impaired performance in bilingual aphasia due to the interaction of executive and lexical deficits. Relatedness measures in category fluency allow for the study of lexical retrieval and executive functions in bilingual people with post‐stroke aphasia
Del Hoyo et al., 2015	Down syndrome	Down syndrome, control	Category fluency (animals), 60s	Troyer et al., 1997	*Cluster size* ^a^ *switches* ^a^	*Total score* ^a^ score over 15 s intervals	Impaired performance in Down syndrome: a combination of lexical (cluster size) and executive (switches) deficits. Category fluency with relatedness measures can be used for the early detection of dementia and amyloidosis in people with Down syndrome
Ehlen et al., 2020	Autism	Autism, control	Category fluency (animals, human features, actions), 120 s	Temporal curve fitting (Ehlen et al., 2016)	*Cluster size* ^a^ switches semantic similarity between consecutive words semantic similarity within clusters semantic category (for human feature fluency) semantic category (for verb fluency)	*Total score* ^a^ cluster duration *switch duration* ^a^ speech velocity orthographic relatedness between consecutive words orthographic relatedness within clusters	Smaller clusters and longer switches suggest smaller semantic networks in ASD, but without differences in the strength of associations in the networks (semantic similarity). Relatedness measures in category fluency can be used to study characteristics of the semantic system in people with autism
Faroqi‐Shah & Milman, 2018	Post‐stroke aphasia (left hemisphere)	Aphasia, control	Category fluency (animals, actions), letter fluency (FAS), 60s	Ledoux et al., 2014	*Number of clusters* ^a^	*Total score* ^a^ perseverations *word frequency* ^a^ *age of acquisition* ^a^ *number of syllables* ^a^ *proportion of light verbs (action fluency)* ^a^	Smaller semantic clusters not only in animal fluency but also in action fluency, suggest lexical retrieval impairments in aphasia. Verbal fluency, especially animal fluency, is sensitive even to mild aphasia, with letter fluency being the least reliant on lexical retrieval and language networks
Grimes et al., 2021	Schizophrenia & schizoaffective	Single cohort (longitudinal)	Category fluency (animals), 60s	Troyer et al., 1997	Cluster size switches	Total score	Lack of longitudinal differences suggests stable verbal fluency abilities in schizophrenia. Relatedness measures in the category fluency are suitable as cognitive measures in schizophrenia, as values do not change unexpectedly
Hall et al., 2017	Specific learning disability	SLD, control	Category fluency (animals, foods), 60s	Animals: Troyer et al., 1997; food: Ross & Murphy, 1999	*Cluster size* ^a^ number of clusters *embedded clusters:total clusters ratio* ^a^ *cluster switches:hard switches ratio* ^a^	*Total score* ^a^ score over 15 s intervals	Lexical‐semantic knowledge was associated with cluster size, while executive control was associated with switches. Clinically, lexical‐semantic deficits in SLD persist into adulthood even in well‐managed cases. Relatedness measures in category fluency allow the study of lexical retrieval and executive functions in bilingual people with SLD
Jaywant et al., 2014	Parkinson's disease	RPD (with vs. without tremor), LPD (with vs. without tremor), control	Category fluency (animals), 60s	Troyer et al., 1997	Number of clusters cluster size *switches* ^a^	Total score	Relatedness‐based measures in category fluency are relatively stable and unimpaired in Parkinson's, with other tasks (e.g., letter fluency, non‐verbal fluency) being more clinically relevant. Relatedness measures in the category fluency can be used to assess language and executive functioning in people with PD
Jiskoot et al., 2023	Genetic FTD	Phenoconverters, non‐converters, control	Category fluency (animals), 60s	Ledoux et al., 2014	*Number of clusters* ^a^ *cluster size* ^a^ *switches* ^a^	*Total score* ^a^ *frequency* ^a^ *age of acquisition* ^a^	Switches and clusters were both associated with executive function, and predictive of symptom onset. Decline in number of switches, cluster size, and number of clusters was associated with cerebellar and frontal neurodegeneration. Relatedness measures in category fluency can be used to study executive functions in people with FTD, and relate to cerebellar brain damage in this population
Johns et al., 2018	Amnestic MCI	aMCI, control	Category fluency (animals), 60s	N/A	Context similarity *order similarity* ^a^ *perceptual similarity* ^a^	Total score *frequency* ^a^ slope of items produced over sessions	Parameters from a semantic representation model can successfully detect differences in memory search processes, and can aid in examining the underlying changes that develop with cognitive impairment. Relatedness measures in category fluency can be used to assess and track impairment in memory search processes in people with MCI
Kiran et al., 2014	Bilingual aphasia (left hemisphere, post‐stroke or TBI)	Bilingual aphasia, bilingual control	Category fluency (animals, clothing, food), 120 s, in 2 language conditions: English, Spanish	Animals: Tschirren et al., 2011; clothing: Rosselli et al., 2002; food: Raboutet et al., 2010	*Cluster size* ^a^ *switches* ^a^	*Total score* ^a^	Cluster size and switches were associated with both aphasia and language group, and indicated impaired lexical retrieval in aphasia while retaining a similar search strategy to controls. Relatedness measures in category fluency lexical retrieval in bilingual people with post‐stroke aphasia
Ku et al., 2021	First‐episode psychosis	Single cohort (tangentiality vs. derailment vs. both vs. neither)	Category fluency (animals), 60s	N/A	Semantic similarity between consecutive words *Coherence‐5* ^a^ *Coherence‐10* ^a^	*Total score* ^a^	Measures of semantic coherence in category fluency can differentiate patients with derailment from those without. Relatedness measures in category fluency can be used as predictive/classification measures in people with psychosis
Leimbach et al., 2020	Parkinson's disease	Pre‐ vs. post‐STN‐DBS surgery, on vs. off STN‐DBS, unoperated	Category fluency (animals, boy's names), letter fluency (FAS), 60s	Troyer et al., 1997	*Semantic cluster size* ^a^ *semantic switches* ^a^ phonological cluster size *phonological switches* ^a^	*Total score* ^a^	Decreased cluster size in category fluency following surgery reflected impairments, while increased cluster size during stimulation reflected altered search strategy. Relatedness measures in category fluency can be used to assess and track impairment in memory search processes in people with Parkinson's disease
Lopez‐Higes et al., 2014	Amnestic MCI	aMCI, control	Category fluency (animals), 60s	Troyer et al., 1997	Cluster size switches	*Total score* ^a^ *score over 15 s intervals* ^a^	Relatedness‐based measures were not statistically significant, and the authors suggest future research with larger sample sizes. Relatedness measures in category fluency can be used as predictive/classification measures in people with psychosis
Lundin, Jones, et al., 2022	Psychosis	Psychosis, control	Category fluency (animals), 60s	Semantic association similarity drop (Word2Vec)	Cluster size *switches* ^a^ switch rate *semantic similarity across switches* ^a^ *semantic similarity within clusters* ^a^	*Total score* ^a^	Lower semantic similarity, both within clusters and across switches, was indicative of disorganized speech and thought soon after psychosis onset, and also correlated with measures of social functioning. Relatedness measures in category fluency can be used as predictive/classification measures in people with psychosis
Mueller et al., 2015	Amnestic MCI	aMCI, control	Category fluency (animals), 60s	Troyer et al., 1997	*Cluster size* ^a^ switches	*Total score* ^a^	Cluster size in category fluency differentiated aMCI from controls, and differences had not previously been reported in a group this young. The study provides support for relatedness measures in category fluency in the targeting of preclinical Alzheimer's disease
Nevado et al., 2021	MCI	MCI, control	Category fluency (animals), 60s	Based on co‐occurrence network‐derived categories (Goñi et al., 2011)	*Cluster size* ^a^ switches *probability of remaining in cluster by word position* ^a^ *probability of remaining in cluster over 15 s intervals* ^a^ Network metrics (network method: Goñi et al., 2011): modularity clustering coefficient betweenness centrality degree average shortest path length diameter	*Total score* ^a^	The authors use co‐occurence measures and graph theory to suggest that semantic networks are preserved in MCI, and that deficits in executive function lead them to be exploited less efficiently in memory search. Computational relatedness measures in category fluency allow the study of semantic network structure, executive functions, and impairments in memory search processes in people with MCI
Nicodemus et al., 2014	Schizophrenia	Schizophrenia, siblings, control	C=Category fluency (animals), 60s	N/A	*N* word sequence coherence (*N* = 1, 2, 3) *average vector length* ^a^ *total vector length* ^a^ semantic similarity to ‘animal’ semantic similarity among all words	*Total score* ^a^	LSA‐derived vector parameters were associated with 3 different genetic polymorphisms in schizophrenia, suggesting that relatedness‐based measures in fluency may offer a more nuanced view of clinical phenotypes. Relatedness measures in category fluency can be used as predictive/classification measures in people with schizophrenia
Nikolai et al., 2018	Subjective cognitive decline	SCD, control	Category fluency (animals, vegetables), 60s	Animals: Troyer et al., 1997; vegetables: Kavé et al., 2007; Kosmidis et al., 2004	Cluster size *switches* ^a^	*Total score* ^a^ *score over 30 s intervals* ^a^	Relatedness‐based measures differed significantly in individuals with subjective cognitive decline, showing their sensitivity to mild deficits which are not captured by traditional neuropsychological tests. Relatedness measures in category fluency can be to assess mild cognitive deficits in people with subjective cognitive decline
Nour et al., 2023	Schizophrenia	Schizophrenia, control	Category fluency (animals), letter fluency (P), 5 min	Communities within semantic similarity network: Louvain agglomerative clustering algorithm (Blondel et al., 2008)	*Semantic similarity between consecutive words* ^a^ *global optimality divergence (semantic)* ^a^ *local optimality divergence (semantic)* ^a^ *cluster size* ^a^ *cluster returns* ^a^ *semantic salience parameter* ^a^ *goal‐induced semantic modulation* ^a^	Global optimality divergence (orthographic) local optimality divergence (orthographic) orthographic salience parameter	The authors use novel and automated computational measures to model conceptual organization in schizophrenia relative to controls, and between participants further associate these measures with neural indicators of cognitive map stabilization in schizophrenia. Computational relatedness measures in category fluency can be used to model semantic network structure and impairments in memory search processes in people with schizophrenia
Pagliarin et al., 2021	Unilateral brain damage	LHD with aphasia, LHD without aphasia, RHD, control	Category fluency (animals), 90s	Troyer et al., 1997	*Clusters* ^a^ *switches* ^a^	*Total score* ^a^ *score over 30 s intervals* ^a^	While the total score suggested that only the LHD group was impaired in category fluency, switching was sensitive to differences in executive function in the RHD group as well. The LHD group exhibited deficits in both clustering and switching, reflecting impaired lexical retrieval and executive function. Relatedness measures in category fluency allow the study of lexical retrieval and executive functions in people with unilateral brain damage, and relate to lesion location
Patra et al., 2020	Bilingual post‐stroke aphasia (left hemisphere)	Bilingual aphasia, bilingual control	Category fluency (animals, fruits/vegetables), 60s	Troyer et al., 1997	Cluster size *switches* ^a^	*Total score* ^a^ within cluster pause duration *switch duration* ^a^ *1st‐RT* ^a^ Sub‐RT *Fluency Difference Score (FDS)* ^a^	Individuals with aphasia were largely impaired only on relatedness‐based measures associated with executive function (switches), and not those associated with lexical retrieval (clusters). This finding was supported by other tasks indicating intact lexical retrieval and impaired executive function. Relatedness measures in category fluency allow the study of lexical retrieval and executive functions in bilingual people with post‐stroke aphasia
Pauselli et al., 2018	Schizophrenia or non‐affective psychosis	Schizophrenia/psychosis (tangentiality vs. derailment vs. both vs. neither), control	Category fluency (animals), 60s	N/A	*Semantic similarity between consecutive words* ^a^ semantic similarity among all words *Coherence‐5* ^a^ *Coherence‐10* ^a^	*Total score* ^a^	Novel measures of semantic coherence in category fluency can differentiate patients with derailment and tangentiality from those without. Relatedness measures in category fluency can be used as predictive/classification measures in people with psychosis
Piras et al., 2019	Schizophrenia	Schizophrenia, control	Category fluency (animals), 60s	Troyer et al., 1997	Cluster size *switches* ^a^	*Total score* ^a^	Lower number of switches in schizophrenia was indicative of impaired executive functioning, while a lack of difference in cluster size was expected due to intact lexical retrieval. Relatedness measures in category fluency allow the study of lexical retrieval and executive functions in people with schizophrenia
Quaranta et al., 2019	Amnestic MCI	aMCI (stable vs. later progressed to AD), control	Category fluency (birds, furniture), 60s	Path Length and Extended Gloss Overlap thresholds (WordNet)	*Clusters* ^a^ *mean path length (WordNet) between consecutive words* ^a^ *Extended Gloss Overlap (WordNet) between consecutive words* ^a^	*Total score* ^a^	WordNet‐derived measures of semantic association between consecutive words reflected differences between individuals with aMCI who later developed dementia and those who did not. Relatedness measures in category fluency can serve as early markers of semantic memory disruption and can be used as predictive/classification measures in people with MCI
Reverberi et al., 2014	AD, svPPA, nfvPPA, bvFTD	AD, svPPA, nfvPPA, bvFTD, control	Category fluency (fruits), 60s	Reverberi et al., 2004, 2006	*Number of categories* ^a^ *switches* ^a^ *switch rate* ^a^ *order index* ^a^ *semantic similarity between consecutive words* ^a^	*Total score* ^a^ *repetitions* ^a^ *out‐of‐category words* ^a^ *average familiarity* ^a^	Relatedness‐based measures and principal component analysis suggested differential impairment in each patient group, with components reflecting different aspects of task performance and cognitive function. Multiple concurrent relatedness measures in category fluency can be used as diagnostic/classification measures, and allow the study of lexical retrieval and executive functions, in people with AD and focal dementias
Rofes et al., 2019	PPA (lvPPA, nfvPPA, svPPA)	svPPA, nfvPPA, lvPPA, control	Category fluency (animals, fruits, vegetables), letter fluency (FAS), 60s	N/A	Semantic similarity between consecutive words	*Total score* ^a^ repetitions inflected words fragments phonological paraphasias neologisms out‐of‐category words age of acquisition concreteness *familiarity* ^a^ frequency imageability length in phonemes orthographic similarity phonological similarity	The relatedness‐based measure used in this study (semantic similarity between consecutive words) was not significantly different between clinical groups. Mean word familiarity within category fluency responses was better than relatedness measures for classifying variants of PPA
Rofes et al., 2020	AD	Single cohort	Category fluency (animals), 60s	Troyer et al., 1997	Cluster size *switches* ^a^ semantic similarity between consecutive words	Total score frequency imageability concreteness familiarity *age of acquisition* ^a^ length in phonemes orthographic similarity phonological similarity	Decreased switching (impaired executive function) and lower age of acquisition among responses (impaired phonological output lexicon) are the primary predictors of performance on category fluency in AD. Relatedness measures in category fluency allow the study of lexical retrieval and executive functions in people with AD
Smith‐Spark et al., 2017	Dyslexia	Dyslexia, control	Category fluency (animals), 60s	Troyer et al., 1997	Cluster size switches	Total score total responses (including errors) total errors	Category fluency and relatedness‐based measures were unimpaired in dyslexia. Phonemic fluency and other measures were more informative. Relatedness measures in category fluency can be used to assess cognitive abilities in people with dyslexia
Tröger et al., 2019	AD, MCI	AD, MCI, control	Category fluency (animals), 60s	Troyer et al., 1997 vs. semantic association threshold (fastText) vs. temporal threshold	Cluster size (Troyer et al., 1997) *switches* (Troyer et al., 1997)^a^ cluster size (semantic association threshold) switches (semantic association threshold) *(semantic) inter‐cluster semantic proximity* ^a^ *(semantic) within cluster semantic proximity* ^a^ cluster size (temporal threshold) *switches (temporal threshold)* ^a^ *(temporal) inter‐cluster semantic proximity* ^a^ (temporal) within cluster semantic proximity *(temporal) semantic proximity difference* ^a^	*Total score* ^a^ repetitions *(semantic) switch duration* ^a^ (semantic) within cluster pause duration (semantic) transition duration difference *(temporal) switch duration* ^a^ *(temporal) within cluster pause duration* ^a^	Results, including novel semantic‐proximity‐based measures, suggest preserved lexical retrieval and impaired executive control in AD, and potentially in MCI. Relatedness measures in category fluency allow the study of lexical retrieval and executive functions in people with AD and MCI
van den Berg et al., 2017	bvFTD, PPA (svPPA, nfvPPA, lvPPA)	svPPA, nfvPPA, lvPPA, bvFTD	Category fluency (animals), letter fluency (DAK/KOM/PGR), 60s	Ledoux et al., 2014	*Cluster size* ^a^ *switches* ^a^ *number of clusters* ^a^ *sum of clustered words* ^a^	*Total score* ^a^	Clustering was significantly associated with memory and language, while switching was significantly associated with executive functioning. Both measures were clinically informative in differentiating subtypes of FTD and PPA. relatedness measures in category fluency can be used as diagnostic/classification measures, and allow the study of lexical retrieval and executive functions, in people with focal dementias
van den Berg et al., 2024	bvFTD, PPA (svPPA, nfvPPA, lvPPA)	bvFTD, svPPA, nfvPPA, lvPPA, control	Category fluency (animals), letter fluency (DAT), 60s	Ledoux et al., 2014	*Cluster size* ^a^ *switches* ^a^ *number of clusters* ^a^	*Total score* ^a^ *frequency* ^a^ *age of acquisition* ^a^ *neighbourhood density* ^a^ *word length* ^a^	Relatedness‐based measures (clustering, switching) differentiated svPPA from other groups, while only word properties differentiated lvPPA and bvFTD. All measures except for word length were further predicted by measures of lexical processing, semantic memory, or executive function. Relatedness measures in category fluency can be used as diagnostic/classification measures, and allow the study of lexical retrieval and executive functions, in people with focal dementias
Voorspoels et al., 2014	Schizophrenia	Schizophrenia, control	Category fluency (animals), 60s	N/A	Geometric representations of semantic relations based on: co‐occurrence in fluency lists proximity and co‐occurance in fluency lists	—	Two novel measures of semantic relations from co‐occurrence and proximity in fluency lists were unable to differentiate schizophrenia from controls. The authors suggest that the novel measures are inappropriate as the underlying representations in schizophrenia may be unimpaired. Relatedness measures in category fluency can be used to assess semantic representations in schizophrenia
Weakley & Schmitter‐Edgecombe, 2014	AD	AD, control	Category fluency (animals), 60s	Troyer et al., 1997	*Cluster size* ^a^ *switches* ^a^ *cluster size over 30 s intervals* ^a^ *switches over 30 s intervals* ^a^ geometric representations of semantic relations based on: proximity in fluency lists	*Total score* ^a^ *score over 30 s intervals* ^a^	Reduced cluster size suggested impaired lexical retrieval in AD, while reduced switching suggested impaired executive function, and semantic organization patterns appeared similar to controls. Relatedness measures in category fluency allow the study of lexical retrieval and executive functions in people with AD
Weakley et al., 2013	MCI	Single‐domain aMCI, multi‐domain aMCI, naMCI, control	Category fluency (animals), 60s	Troyer et al., 1997	Cluster size *switches* ^a^ cluster size over 30 s intervals switches over 30 s intervals	*Total score* ^a^ score over 30 s intervals	Reduced switching, linked to executive functioning, was the strongest predictor of reduced task performance and differentiates individuals with multidomain MCI and naMCI. Relatedness measures in category fluency can be used as predictive/classification measures, and allow the study of lexical retrieval and executive functions, in people with MCI
Zemla & Austerweil, 2019	AD	AD, control	Category fluency (animals), 60s (3+ lists per participant)	N/A	Network metrics (network method: U‐INVITE, Zemla et al., 2016): *number of nodes* ^a^ *diameter* ^a^ *density* ^a^ *average shortest path length* ^a^ clustering coefficient *smallworldness* ^a^ *node degree* ^a^	*Total score* ^a^ *perseverations* ^a^	A novel method of network generation from fluency lists suggested that semantic networks in AD are more connected and more randomly distributed than those of controls. This relatedness‐based network method improves classification relative to traditional fluency measures. Relatedness measures in category fluency allow the study of semantic network structure in people with AD

^a^ Variables for which significant between‐group differences, significant correlations or high importance in a predictive model were found.

### What tasks employing relatedness‐based measures have been reported in adults with language disorders?

All eligible studies (*N* = 38) included in the present review made use of the category fluency task, paired with analytical techniques that investigated relatedness in meaning between the items produced by participants. These relatedness measures consisted largely of sequence‐level fluency measures such as clustering and switching (described in more detail in Section ‘What analysis methods are used in these tasks?’). Three studies among those additionally made use of measures of relatedness in meaning between items on phonological or letter fluency (Leimbach et al., 2020; Nour et al., 2023; Rofes et al., 2019). Three further studies made use of a clustering method that allows for either semantically or phonologically defined clusters in both category and letter fluency (Ledoux et al., 2014), without explicitly mentioning the degree to which each association type was identified among responses to the two tasks (Faroqi‐Shah & Milman, 2018; van den Berg et al., 2017, 2024).

No studies were identified in which performance on a task led to an investigation of relatedness in meaning between items presented as part of the experimental paradigm, or in which participants gave relatedness judgements between items.

### What types of language disorders and neurological pathologies are discussed?

The most commonly discussed language disorder among identified studies was post‐stroke aphasia, with 7 studies (18.4%) focusing on this clinical population (Bose et al., 2017, 2022; Carpenter et al., 2021; Faroqi‐Shah & Milman, 2018; Kiran et al., 2014; Pagliarin et al., 2021; Patra et al., 2020). Of these, 3 (7.9%) specifically focus on bilingual post‐stroke aphasia (Carpenter et al., 2021; Kiran et al., 2014; Patra et al., 2020), and 2 (5.3%) additionally include participants with aphasia following TBI (Carpenter et al., 2021; Kiran et al., 2014). Similarly, there were 7 studies (18.4%) which involved individuals with mild cognitive impairment (MCI; Johns et al., 2018; Lopez‐Higes et al., 2014; Mueller et al., 2015; Nevado et al., 2021; Quaranta et al., 2019; Tröger et al., 2019; Weakley et al., 2013), and 8 studies (21.1%) which involved individuals with either schizophrenia, schizoaffective disorder, or psychosis (Grimes et al., 2021; Ku et al., 2021; Lundin, Jones, et al., 2022; Nicodemus et al., 2014; Nour et al., 2023; Pauselli et al., 2018; Piras et al., 2019; Voorspoels et al., 2014). Five studies (13.2%) involved individuals with AD (Reverberi et al., 2014; Rofes et al., 2020; Tröger et al., 2019; Weakley & Schmitter‐Edgecombe, 2014; Zemla & Austerweil, 2019). Four studies (10.5%) involved individuals with variants of primary progressive aphasia (PPA; Reverberi et al., 2014; Rofes et al., 2019; van den Berg et al., 2017, 2024) while 3 (7.9%) included individuals with variants of frontotemporal dementia (FTD; Jiskoot et al., 2023; van den Berg et al., 2017, 2024). Two studies (5.3%) involved individuals with Parkinson's disease (Jaywant et al., 2014; Leimbach et al., 2020). One study each (2.6%) was identified for individuals with multiple sclerosis (Altun et al., 2024), specific learning disability (SLD, Hall et al., 2017), Down syndrome (Del Hoyo et al., 2015), autism (Ehlen et al., 2020), subjective cognitive decline (Nikolai et al., 2018), unilateral post‐stroke brain damage without aphasia (Pagliarin et al., 2021), and dyslexia (Smith‐Spark et al., 2017). Note that the percentages above do not add up to 100 as several studies investigated more than one of the stated populations.

### What types of items are used in these tasks, and how are those items selected?

As no studies were identified for which relatedness was investigated between predetermined items, there were likewise no item selection methods revealed by the present review. While the identified studies made use of various semantic constraints in category fluency (e.g., animals, vegetables, furniture), item‐level selection methods were not applicable to any of the identified relatedness‐based tasks. Specifically, of the 38 identified studies, 36 (94.7%) used the category of ‘animals’ in the category fluency task, while Quaranta et al. (2019) used both ‘birds’ and ‘furniture’, and Reverberi et al. (2014) used ‘fruits’. Of the 36 papers which used the category of ‘animals’, 9 studies (25%) accompanied the ‘animal’ category with additional semantic conditions (Carpenter et al., 2021; Ehlen et al., 2020; Faroqi‐Shah & Milman, 2018; Hall et al., 2017; Kiran et al., 2014; Leimbach et al., 2020; Nikolai et al., 2018; Patra et al., 2020; Rofes et al., 2019). The vast majority of studies employed the category fluency task with a time limit of 60 s, with only four articles identified for which a longer time limit was used: 90 s (Pagliarin et al., 2021), 120 s (Ehlen et al., 2020; Kiran et al., 2014) and 5 min (Nour et al., 2023). No identified study stated a theoretical or experimental reason for their choice of time limit, other than the fact that particular time limits are indicated in the verbal fluency subtest of different research batteries (e.g., Bose et al., 2017; Pagliarin et al., 2021).

### What analysis methods are used in these tasks?

Of the 38 category fluency studies identified, 31 (81.6%) made use of relatedness‐based measures derived from clustering and switching behaviour (Altun et al., 2024; Bose et al., 2017, 2022; Carpenter et al., 2021; Del Hoyo et al., 2015; Ehlen et al., 2020; Faroqi‐Shah & Milman, 2018; Grimes et al., 2021; Hall et al., 2017; Jaywant et al., 2014; Jiskoot et al., 2023; Kiran et al., 2014; Leimbach et al., 2020; Lopez‐Higes et al., 2014; Lundin, Jones, et al., 2022; Mueller et al., 2015; Nevado et al., 2021; Nikolai et al., 2018; Nour et al., 2023; Pagliarin et al., 2021; Patra et al., 2020; Piras et al., 2019; Quaranta et al., 2019; Rofes et al., 2020; Smith‐Spark et al., 2017; Tröger et al., 2019; van den Berg et al., 2017, 2024; Weakley et al., 2013; Weakley & Schmitter‐Edgecombe, 2014). Seven studies (18.4%) did not make use of clustering or switching measures and are discussed later in this review (Johns et al., 2018; Ku et al., 2021; Nicodemus et al., 2014; Piras et al., 2019; Rofes et al., 2019; Voorspoels et al., 2014; Zemla & Austerweil, 2019).

A number of different semantic clustering methods were used in determining clusters of related items. The method established by Troyer et al. (1997) for animal fluency was most common, being used by 20 of the 31 studies which used clustering and switching measures (64.5%; Altun et al., 2024; Bose et al., 2017, 2022; Carpenter et al., 2021; Del Hoyo et al., 2015; Grimes et al., 2021; Hall et al., 2017; Jaywant et al., 2014; Leimbach et al., 2020; Lopez‐Higes et al., 2014; Mueller et al., 2015; Nikolai et al., 2018; Pagliarin et al., 2021; Patra et al., 2020; Piras et al., 2019; Rofes et al., 2020; Smith‐Spark et al., 2017; Tröger et al., 2019; Weakley et al., 2013; Weakley & Schmitter‐Edgecombe, 2014). Four of the 31 studies which used cluster analysis (10.5%) used the method proposed by Ledoux et al. (2014), in which either semantic or phonological clusters in the style of Troyer et al. (1997) are allowable in both animal and letter fluency (Faroqi‐Shah & Milman, 2018; Jiskoot et al., 2023; van den Berg et al., 2017, 2024). Four further studies of the 31 that used cluster analysis (10.5%) used other subcategory‐based clustering methods analogous to those of Troyer et al. (1997), primarily for use in categories other than animals (Hall et al., 2017; Kiran et al., 2014; Nikolai et al., 2018; Reverberi et al., 2014).

Clustering methods were also identified which did not rely on categories or predetermined lists of classifiers. Ehlen et al. (2020) made use of a temporal curve fitting model^1^ established in an earlier paper by the same group (Ehlen et al., 2016), complemented by corpus‐driven inter‐item semantic association measures. A similarity‐based method derived from word embedding models was used by two studies (Lundin, Jones, et al., 2022; Tröger et al., 2019). Nevado et al. (2021) generated semantic networks from fluency data using a method based on co‐occurrence (Goñi et al., 2011), defining clusters based on subdivisions within this network, while Quaranta et al. (2019) based clusters on participant‐specific thresholds for Path Length and Extended Gloss Overlap, metrics derived from WordNet (Fellbaum, 1998, 2005). Nour et al. (2023) examined clustering behaviour in the context of communities in a semantic similarity network, with communities determined by a Louvain agglomerative clustering algorithm^2^ (Blondel et al., 2008).

Two of the 31 studies that used cluster analysis (6.5%) compared multiple clustering methods within the same task (Leimbach et al., 2020; Tröger et al., 2019). Tröger et al. (2019) used the method defined by Troyer et al. (1997) in addition to clusters based on both semantic association thresholds and temporal thresholds, while Leimbach et al. (2020) analysed both animal and letter fluency using both the semantic and phonological clustering methods defined by Troyer et al. (1997).

Among the studies for which clustering and switching were not considered, a number of analytical methods were employed to measure relatedness in meaning between items. To classify individuals with amnestic MCI relative to controls, Johns et al. (2018) used a maximum likelihood estimation model^3^ considering parameters for context similarity and order similarity between consecutive words (both from the BEAGLE semantic space, Jones & Mewhort, 2007), perceptual similarity (Johns & Jones, 2012), and frequency from a Wikipedia corpus (Jones & Mewhort, 2007). CoVec, an automated tool (Covington, 2016) was used by two studies to differentiate psychosis patients with different speech patterns, considering sliding 5 and 10‐word windows to establish measures of coherence (Coherence‐5 and Coherence‐10, Ku et al., 2021; Pauselli et al., 2018). Nicodemus et al. (2014) differentiated schizophrenia patients from controls and established correlations with specific genetic mutations, through the use of latent semantic analysis (LSA) derived fluency measures (Landauer et al., 1998). Voorspoels et al. (2014) explored the use of geometric representations of group‐level and subsample‐level semantic relations based on both proximity and co‐occurrence of items in schizophrenic patients' category fluency lists. Rofes et al. (2019) used random forests to classify PPA variants based on total score, 6 error types, and 9 word properties, including LSA‐derived semantic association measures (with a similar methodology to Rofes et al. (2020), which did consider cluster and switch behaviour). Zemla and Austerweil (2019) generated semantic networks from fluency lists based on a censored random walk methodology, differentiating the networks of individuals with AD from those of controls on six network metrics: number of nodes, diameter (D), density, average shortest path length (ASPL), smallworldness (S), and node degree.

Network methodologies were additionally employed by four of the studies which did consider clusters and switches. Quaranta et al. (2019) investigated the mean path length between consecutive fluency items based on their node proximity in WordNet, a lexical database of semantic relations (Fellbaum, 1998, 2005). Weakley and Schmitter‐Edgecombe (2014) used multidimensional scaling to generate group‐level geometric representations of semantic relations between words based on their proximity in fluency lists. Nevado et al. (2021) generated semantic networks for individuals with MCI and controls based on the methods of Goñi et al. (2011), comparing network metrics such as modularity (Q), clustering coefficient (CC), betweenness centrality (BC), degree, ASPL and D. Nour et al. (2023) modelled fluency responses of individuals with schizophrenia as trajectories through semantic spaces, investigating novel computational measures which measure the degree to which fluency lists deviate from an ‘optimal’ path through the semantic space.

Lastly, while all identified studies made use of relatedness‐based measures, 10 (26.3%) supplemented these measures with additional word‐level differences between items (e.g., word properties, orthographic distances; Ehlen et al., 2020; Faroqi‐Shah & Milman, 2018; Jiskoot et al., 2023; Johns et al., 2018; Leimbach et al., 2020; Nour et al., 2023; Reverberi et al., 2014; Rofes et al., 2019, 2020; van den Berg et al., 2024). For an overview of all relatedness measures derived from verbal fluency in the identified studies, in addition to those for which significant between‐group differences, significant correlations, or high importance in predictive models were found, please refer to Table 2.

### Theoretical contributions

The identified studies use relatedness‐based measures to make theoretical contributions to our understanding of category fluency performance, particularly in differentiating lexical and executive task demands. Twenty‐one studies (55.3%) considered relatedness‐based measures based on clustering behaviour as measures of lexical retrieval or verbal memory (Altun et al., 2024; Bose et al., 2017; Bose et al., 2022; Carpenter et al., 2021; Del Hoyo et al., 2015; Ehlen et al., 2020; Faroqi‐Shah & Milman, 2018; Hall et al., 2017; Kiran et al., 2014; Lopez‐Higes et al., 2014; Mueller et al., 2015; Pagliarin et al., 2021; Patra et al., 2020; Piras et al., 2019; Reverberi et al., 2014; Rofes et al., 2020; Tröger et al., 2019; van den Berg et al., 2017, 2024; Weakley et al., 2013; Weakley & Schmitter‐Edgecombe, 2014). Five of these 21 studies (23.8%) further showed significant associations between clustering and separate measures of linguistic cognition (Hall et al., 2017; Mueller et al., 2015; Patra et al., 2020; van den Berg et al., 2017, 2024). Similarly, 22 studies (57.9%) consider relatedness‐based measures based on switching behaviour as measures of executive function (Altun et al., 2024; Bose et al., 2017, 2022; Carpenter et al., 2021; Del Hoyo et al., 2015; Ehlen et al., 2020; Faroqi‐Shah & Milman, 2018; Hall et al., 2017; Jiskoot et al., 2023; Kiran et al., 2014; Lopez‐Higes et al., 2014; Mueller et al., 2015; Pagliarin et al., 2021; Patra et al., 2020; Piras et al., 2019; Reverberi et al., 2014; Rofes et al., 2020; Tröger et al., 2019; van den Berg et al., 2017, 2024; Weakley et al., 2013; Weakley & Schmitter‐Edgecombe, 2014). Seven of these 22 studies (31.8%) further showed significant associations between switching and separate measures of executive function (Hall et al., 2017; Jiskoot et al., 2023; Mueller et al., 2015; Patra et al., 2020; van den Berg et al., 2017, 2024; Weakley et al., 2013).

Some of the identified studies further use relatedness‐based measures to make theoretical contributions to our understanding of clinical conditions. All 7 studies investigating post‐stroke aphasia used clustering and switching to differentiate executive and lexical deficits in this population (Bose et al., 2017, 2022; Carpenter et al., 2021; Faroqi‐Shah & Milman, 2018; Kiran et al., 2014; Pagliarin et al., 2021; Patra et al., 2020). There were further insights into the effects of language dominance on performance in bilingual aphasia (e.g., Kiran et al., 2014), and the appropriateness of different categories of semantic fluency in the assessment of lexical abilities and disease severity in this population (Faroqi‐Shah & Milman, 2018).

In MCI, relatedness‐based measures are suggested as early markers of disruptions to either semantic memory or search strategy, which may offer predictive value in determining those at risk of developing AD (Johns et al., 2018; Mueller et al., 2015; Nevado et al., 2021; Quaranta et al., 2019; Tröger et al., 2019; Weakley et al., 2013). In AD itself, various measures are used to differentiate executive, lexical retrieval and phonological output deficits (Reverberi et al., 2014; Rofes et al., 2020; Tröger et al., 2019; Weakley & Schmitter‐Edgecombe, 2014), to differentiate AD from other conditions characterized by dementia (Reverberi et al., 2014; Tröger et al., 2019), or to gain insights into the structure of semantic memory (Zemla & Austerweil, 2019).

In schizophrenia and psychosis, relatedness‐based measures in category fluency were used as indicators of disorganized thought and speech (Ku et al., 2021; Lundin, Jones, et al., 2022; Pauselli et al., 2018), and also to suggest longitudinal stability in task performance (Grimes et al., 2021). They further contributed to a more nuanced perspective of clinical phenotypes associated with different biological markers (Nicodemus et al., 2014; Piras et al., 2019), and to competing views regarding the underlying structure of semantic organization in this population (Nour et al., 2023; Voorspoels et al., 2014).

Lastly, 2 of the 38 identified studies (5.3%) make detailed theoretical contributions to our understanding of the neural architecture supporting language and task performance. Jiskoot et al. (2023) explored the relationship between longitudinal changes in fluency measures with grey matter (GM) volume loss in FTD and found that decreases in cluster size, clusters and switches were associated with GM volume loss in the cerebellum, frontal areas, insular cortex and putamen. They further found associations between the frequency and age of acquisition of words and GM losses in the cerebellum and temporal areas (Jiskoot et al., 2023). In individuals with schizophrenia, Nour et al. (2023) found that the influence of semantic similarity on behaviour in verbal fluency is associated with hippocampal ripple bursts, a MEG‐derived measure of structured memory replay.

For a more detailed description of all other theoretical contributions offered by each of the identified studies to our understanding of task performance, clinical deficits and the architecture of language, please refer to the ‘Theoretical contributions’ column of Table 2.

## DISCUSSION

The goal of this scoping review was to comprehensively explore the landscape of relatedness‐based measures as they are utilized in populations with language disorders. We sought to address several key questions, namely the types of tasks employed, the language disorders under investigation, the types of items used and the analytical methods applied. This work is significant because, to date, no extensive review or overview of relatedness‐based measures in these populations has been presented. Tasks that employ these measures provide a unique opportunity to leverage novel computational methods from network science, potentially enabling more in‐depth investigations of mild language impairments and the underlying nature of the lexico‐semantic organization.

The results of this review revealed a predominant use of category fluency tasks with relatedness‐based measures in linguistic populations, with all identified studies (*N* = 38) making use of this experimental paradigm. Despite a search strategy designed to encapsulate other tasks using relatedness‐based measures, no studies were found where task performance prompted an exploration of the semantic relatedness between experimenter‐presented items, nor where participants provided relatedness judgements between them. On the one hand, this finding underscores the utility of category fluency in assessing lexico‐semantic processing across various clinical populations, and likely reflects its ease of use, short administration time and frequent inclusion in both neuropsychological and linguistic assessment batteries (Rabin et al., 2005; Rofes et al., 2017, 2021; Rook et al., 2023; Strauss et al., 2006). However, the exclusive reliance on category fluency tasks has the potential to limit the scope of insights into lexico‐semantic deficits. Indeed, fluency tasks also require non‐linguistic aspects, such as executive functioning, with performance dependent on both associative and controlled processes (Rofes et al., 2023; Shao et al., 2014). This raises the issue of ‘task impurity’, which highlights the fact that tasks might not depend on single cognitive processes (Rabbitt, 1997). Hence, the results obtained with these tasks can be to a certain extent affected, particularly if patients have impairments in executive processes of shifting, updating and monitoring (Rofes et al., 2023). From this perspective, it is notable that among the identified category fluency studies, several complemented relatedness‐based measures with additional word‐level differences between items (i.e., Ehlen et al., 2020; Faroqi‐Shah & Milman, 2018; Jiskoot et al., 2023; Johns et al., 2018; Leimbach et al., 2020; Nour et al., 2023; Reverberi et al., 2014; Rofes et al., 2019, 2020; van den Berg et al., 2024). This may offer the opportunity to disentangle the degree to which presumed lexico‐semantic relationships between items are influenced by additional factors, linguistic or otherwise. Similarly, 22 studies used different relatedness‐based measures, such as clustering and switching, to examine the relative contribution of distinct cognitive deficits to task performance (e.g., lexical retrieval, executive function), further contributing to our theoretical understanding of category verbal fluency and the interpretation of its results (e.g., Hall et al., 2017; van den Berg et al., 2024).

Notably, only four studies applied a time limit exceeding 60 s in the category fluency task (Ehlen et al., 2020; Kiran et al., 2014; Nour et al., 2023; Pagliarin et al., 2021). Initial stages of verbal fluency tasks are associated with rapid, semi‐automatic retrieval, while later stages involve slower, effortful semantic memory retrieval, with interval‐level fluency scores being clinically predictive (Amunts et al., 2021; Fernaeus & Almkvist, 1998; Jacobs et al., 2021). The prevalence of 60‐s limits may represent a missed opportunity clinically if this effect becomes more pronounced with longer administration times, particularly in fatigue‐prone groups such as low‐grade glioma patients (Facque et al., 2022). Experimentally, longer administration times would also provide more data points for relatedness‐based measures, potentially enhancing insights into lexico‐semantic organization and the cognitive processes at play throughout the task. Future research should seek a balance between the increase in lexico‐semantic data that comes from 5‐min time limits as in Nour et al. (2023) and the more clinically expedient 60‐s time limits seen in the majority of the literature.

Another aspect is that, while category fluency tasks capture semantic relatedness indirectly through clustering, switching, and other semantic‐search‐related behaviours (Hills et al., 2012; Ovando‐Tellez, Benedek, et al., 2022), other paradigms explicitly probing relatedness, such as relatedness judgement tasks (e.g., Ovando‐Tellez, Kenett, et al., 2022), were notably absent. Semantic networks derived from relatedness judgement tasks have been utilized in studies involving healthy populations, particularly in research focusing on creativity (e.g., Benedek et al., 2017; He et al., 2021), and the lack of comparable paradigms in clinical populations may present a notable void in the literature. Preliminary findings by the authors have indicated that network metrics obtained from relatedness judgement tasks may exhibit significant disparities between individuals who have undergone awake brain surgery and healthy controls (Gaudet et al., 2023), which may indicate mild lexico‐semantic impairments beyond the detection of traditional tests. The lack of tasks in which relatedness between predetermined task items was investigated also precluded this review from addressing the types of stimuli used, as well as their selection methods.

A diverse range of clinical populations were addressed across the identified studies, with post‐stroke aphasia being the most frequently studied language disorder (*n* = 7), in addition to many studies investigating MCI (*n* = 7) and schizophrenia (*n* = 8). Along with the inclusion of studies addressing conditions such as autism, Down syndrome, and specific learning disabilities, this diversity may highlight not only the multifaceted nature of lexico‐semantic processing deficits but also the importance of investigating relatedness‐based measures across different clinical populations. Owing in part to the previously mentioned ability for relatedness‐based measures to provide insight into the nature of task performance differences, the identified papers demonstrated the utility of these measures in describing, classifying and even predicting clinical conditions from a theoretically cohesive perspective (e.g., Bose et al., 2022; Jiskoot et al., 2023; Tröger et al., 2019; van den Berg et al., 2024). The prevalence of novel methodologies within psychiatric research, particularly in the study of schizophrenia, schizoaffective disorder and psychosis is also of particular interest. Of the 8 such studies retrieved, 6 (75%) made use of either cosine similarities, co‐occurrence data, or fluency‐derived network metrics (Ku et al., 2021; Lundin, Jones, et al., 2022; Nicodemus et al., 2014; Nour et al., 2023; Pauselli et al., 2018; Voorspoels et al., 2014), while no such methods were found in the literature for post‐stroke aphasia. While this may in part be due to the more severe nature of linguistic deficits in post‐stroke aphasia relative to schizophrenia (Little et al., 2019), computational techniques that rely on vector space models and network science offer a level of automation not possible with traditional scoring methods, as well as a potential solution to issues of inter‐rater reliability (Kim et al., 2019). As such, their use in post‐stroke aphasia research may offer an expedited, computationally robust and more reliable analysis of fluency responses.

Of particular note is the lack of relatedness‐based measures being used for individuals with brain tumours, as just one study was returned for which an individual with a brain tumour was included (among a bilingual aphasic study population, Carpenter et al., 2021). Lexico‐semantic deficits are prevalent among individuals who have undergone surgery for brain tumours, especially those with high‐grade gliomas (Campanella et al., 2009), and often manifest as difficulties in retrieving words, anomia, semantic paraphasias and neologisms (Goodglass & Wingfield, 1997; Satoer et al., 2018; Whitworth et al., 2014). It is possible that studies employing relatedness‐based measures paired with novel computational methods in individuals with brain tumours could offer additional insights into the nature of not only these deficits, but the neurocognitive mechanisms which underlie them. Importantly, to date none of the studies we reviewed directly studied the predictive value of relatedness‐based measures to classify individuals with different brain aetiologies. Therefore, a relevant question for future studies could be assessing whether any fluency measure (or any relatedness‐based measure that can be extracted from either these tasks or other lexico‐semantic tasks) has sufficient power to tell apart individuals with different brain aetiologies. In our opinion, this sets out to be a difficult question since, from what we see in this review, fluency tasks can for example be used to assess impairments in lexico‐semantic processing and in executive functions, and impairments in these cognitive processes can affect people with different neurological conditions similarly (e.g., Jaywant et al., 2014; Rofes et al., 2019; van den Berg et al., 2024).

The analysis methods employed in the identified studies varied widely, ranging from the more prevalent traditional measures of clustering and switching (e.g., Mueller et al., 2015) to advanced computational techniques such as semantic network analysis and measures of inter‐item coherence (e.g., Ku et al., 2021; Zemla & Austerweil, 2019). While traditional measures like clustering and switching provide valuable insights into semantic organization and cognitive processes (Thiele et al., 2016), the integration of computational approaches may offer novel avenues for understanding complex relationships between items in semantic memory. Notably, some studies employed multiple analysis methods within the same task, highlighting the potential for complementary insights from different analytical approaches (e.g., Nevado et al., 2021; Tröger et al., 2019). The clinical populations, analytical methods and conclusions of the identified studies may be too heterogeneous to draw definitive conclusions from. However, the authors hope that the present review can provide some guidance for future research depending on both target populations and the cognitive or lexico‐semantic processes of interest.

Of note is the relative lack of identified studies that considered the neural correlates of performance on tasks with relatedness‐based measures in clinical linguistic populations. In a longitudinal investigation of genetic FTD, Jiskoot et al. (2023) found that a decline in switches, cluster size and number of clusters was associated with neurodegeneration in cerebellar and frontal regions, in addition to neural findings related to word properties. In schizophrenia, Nour et al. (2023) associated novel measures modelling conceptual organization with neural indicators of cognitive map stabilization. It is perhaps a promising sign of a trend in the literature that both of these papers were from the tail end of our search period. However, investigations of neural correlates were absent in the remainder of the identified studies, with the potential exception of studies whose experimental groups were divided based on laterality (e.g., Jaywant et al., 2014; Pagliarin et al., 2021). Tasks which investigate the level of relatedness in meaning between distinct concepts or words can provide unique insight into not only the organization of items, but also the strength of the relationships between these items, and the processes involved in making those associations (e.g., Nevado et al., 2021; Okruszek et al., 2013; Ovando‐Tellez, Benedek, et al., 2022). When paired with neuroimaging techniques such as functional magnetic resonance imaging (fMRI), they can also inform our understanding of the lexico‐semantic neural substrates. For example, judgements based on both taxonomic and thematic associations were found in an fMRI study to equivalently engage the same core network, consisting of the anterior temporal lobe, superior temporal sulcus and ventral prefrontal cortex (Jackson et al., 2015). In an investigation of search behaviour, fMRI concurrent with a category fluency task has further shown both hippocampal and cerebellar activation during the switch from one cluster of related responses to another (Lundin, Brown, et al., 2022), while others propose the involvement of executive frontal lobe processes, the occipital lobe, and the interaction of multiple large‐scale brain networks in similar semantic search behaviour (Birn et al., 2010; Li et al., 2017; Ovando‐Tellez, Benedek, et al., 2022; Troyer et al., 1998). Assessing the neural correlates of performance on tasks with relatedness‐based measures might further elucidate the neuroarchitecture supporting critical elements of linguistic cognition, particularly in populations where neuroimaging data may already exist.

One of the limitations of this study is that the review was restricted to studies published within 11‐year timeframe. This was primarily due to the recency of the novel methodologies of interest, and also due to the broad search strategy found to be necessary in capturing all relevant studies. It remains possible that this could have excluded earlier notable works in the field. There was also a lack of search terms specifically targeting psychiatric research, despite the inclusion of more general terms that could be expected to encapsulate such articles. Lastly, the heterogeneity of methodologies and outcome measures across studies limits the comparability of findings and generalizability of conclusions. Future research in populations with language disorders should address the aforementioned gaps in the literature regarding paradigms that explicitly probe relatedness between items (e.g., relatedness judgement tasks), the use of relatedness‐based measures in individuals with brain tumours, and the continued development of novel computational and automated techniques.

## CONCLUSION

This scoping review sought to provide a comprehensive synthesis of tasks employing relatedness‐based measures in populations with language disorders, shedding light on commonly used paradigms and analytical methods over the last 11 years. Relatedness‐based measures hold promise as valuable tools for assessing and understanding lexico‐semantic deficits in clinical populations.

## AUTHOR CONTRIBUTIONS

**Logan A. Gaudet:** Conceptualization; data curation; formal analysis; investigation; methodology; project administration; visualization; writing – original draft; writing – review and editing. **Lena Rybka:** Investigation; methodology; writing – review and editing. **Emmanuel Mandonnet:** Investigation; supervision; writing – review and editing. **Emmanuelle Volle:** Investigation; writing – review and editing. **Marion Barberis:** Investigation; writing – review and editing. **Roel Jonkers:** Conceptualization; investigation; methodology; supervision; writing – review and editing. **Adrià Rofes:** Conceptualization; investigation; methodology; supervision; writing – review and editing.

## FUNDING INFORMATION

AR received funding from the Dutch Research Council (SSH Open Competition XS Pilot—406.XS.01.050).

## CONFLICT OF INTEREST STATEMENT

All authors declare no conflict of interest.

## PRE‐REGISTRATION DETAILS

PROSPERO 2024 CRD42024508094. Available from: https://www.crd.york.ac.uk/prospero/display_record.php?ID=CRD42024508094.

## Supporting information


Table S1.

## Data Availability

Data sharing is not applicable to this article as no new data were created or analysed in this study.

## Footnotes

^1^
Described by the authors as a ‘fused bousfieldian function for modelling word production’, consecutive words were plotted over time, and curve sections with steeper than predicted slopes were defined as temporal clusters (Ehlen et al., 2016).

^2^
Described by the authors as a ‘data driven agglomerative clustering procedure’ (Nour et al., 2023), semantic similarities (cosine distances) were used to cluster items using a method defined in more detail by Blondel et al. (2008).

^3^
Maximum likelihood estimation is a statistical method (Myung, 2003) which was used to fit model parameters to participant responses. The authors state that a grid‐search algorithm was used (Johns et al., 2018).
