# Supplementary material for: Leveraging relatedness‐based measures in people with language disorders: A scoping review
Source: J Neuropsychol. 2024 Dec 16;19(2):299–337. doi: 10.1111/jnp.12405 (PMC12166655; doi:10.1111/jnp.12405)
Supplement: Supplementary file 1 — Table S1. [file JNP-19-299-s001.docx]

**Table S1** Search string used for article retrieval in each database

| Database | Search String | Limit |
| --- | --- | --- |
| PubMed (<https://pubmed.ncbi.nih.gov/>) | ("Language Disorders"[Mesh] OR "Aphasia"[Mesh] OR "Brain Neoplasms"[Mesh] OR "Communication Disorders"[Mesh]) AND ("Word Association Tests"[Mesh] OR "verbal fluency"[tiab] OR “semantic fluency”[tiab] OR “category fluency”[tiab] OR ("cluster*"[tiab] AND "switch*"[tiab]) OR “semantic network*”[tiab] OR (("related*"[tiab] OR "association"[tiab] OR “associative”[tiab] OR "semantic"[tiab]) AND (“task”[tiab] OR “test”[tiab] OR “judgment”[tiab] OR “judgement”[tiab] OR “evaluation”[tiab]))) | October 2013 - September 2024 |
| EMBASE  (<https://www.embase.com/>) | ('language disability'/exp OR 'aphasia'/exp OR 'brain tumor'/exp OR 'communication disorder'/exp) AND ('word association tests'/exp OR 'verbal fluency':ti,ab OR 'semantic fluency':ti,ab OR 'category fluency':ti,ab OR ('cluster*':ti,ab AND 'switch*':ti,ab) OR 'semantic network*':ti,ab OR (('related*':ti,ab OR 'association':ti,ab OR 'associative':ti,ab OR 'semantic':ti,ab) AND ('task':ti,ab OR 'test':ti,ab OR 'judgment':ti,ab OR 'judgement':ti,ab OR 'evaluation':ti,ab))) | October 2013 - September 2024 |
| Web of Science  ([clarivate.com/products/web-of-science/](http://clarivate.com/products/web-of-science/)) | TS=('language disability' OR 'aphasia' OR 'brain tumor' OR 'communication disorder') AND (TS=('word association tests' OR 'verbal fluency' OR 'semantic fluency' OR 'category fluency' OR ('cluster*' AND 'switch*') OR 'semantic network*') OR TS=('related*' OR 'association' OR 'associative' OR 'semantic') AND (TS=('task' OR 'test' OR 'judgment' OR 'judgement' OR 'evaluation'))) | October 2013 - September 2024 |
| PsycInfo  (<https://apa.org/pubs/databases/psycinfo/>) | (SU "Language Disorders" OR SU "Aphasia" OR SU "Brain Neoplasms" OR SU "Communication Disorders") AND (SU "Word Associations" OR AB “verbal fluency” OR TI “verbal fluency” OR AB “semantic fluency” OR TI “semantic fluency” OR AB “category fluency” OR TI “category fluency” OR ((AB "cluster*" OR TI “cluster*”) AND (AB "switch*" OR TI “switch*”)) OR AB “semantic network*” OR TI “semantic network*” OR ((AB "related*" OR TI “related*” OR AB "association" OR TI “association” OR AB “associative” OR TI “associative” OR AB "semantic" OR TI “semantic”) AND (AB “task” OR TI “task” OR AB “test” OR TI “test” OR AB “judgment” OR TI “judgment” OR AB “judgement” OR TI “judgement” OR AB “evaluation” OR TI “evaluation”))) | October 2013 - September 2024 |
